# Addressing HIV Misconceptions among Heterosexual Black Men and Communities in Ontario

**DOI:** 10.3390/healthcare11070997

**Published:** 2023-03-31

**Authors:** Egbe B. Etowa, Josephine Pui-Hing Wong, Francisca Omorodion, Josephine Etowa, Isaac Luginaah

**Affiliations:** 1 Daphne Cockwell Health Sciences Complex, Toronto Metropolitan University, Toronto, ON M5B 2K3, Canada; 2 Department of Sociology, Anthropology and Criminology, University of Windsor, Windsor, ON N9B 3P4, Canada; 3 School of Nursing, University of Ottawa, Ottawa, ON K1N 6N5, Canada; 4 Department of Geography, University of Western Ontario, London, ON N6A 3K7, Canada

**Keywords:** Black men, heterosexuality, HIV misconceptions, social determinants

## Abstract

Background. Black males accounted for 19.7% of all the new HIV diagnoses in Canada in 2020, yet Black people make up only 4.26% of the population. Persistent misconceptions about modes of HIV transmission need to be addressed to reduce the relatively high HIV prevalence among Black men. We described the HIV misconceptions held by some HBM in Ontario. We also identified the social determinants that are protective versus risk factors for HIV misconceptions among heterosexual Black men (HBM) in Ontario with a view to building evidence-based strategies for strengthening HIV prevention and stigma reduction among HBM and their communities in Ontario. Methods. We report quantitative findings of the weSpeak study carried out among HBM in four cities (Ottawa, Toronto, London, and Windsor) in Ontario. Sample size was 866 and sub-samples were: Ottawa (n = 210), Toronto (n = 343), London (n = 157), and Windsor (n = 156). Data were collected with survey questionnaire. The outcome variable, HIV misconception score ranging from 1 to 18, was measured by the number of statements on the HIV Knowledge Questionnaire with incorrect answers. We included three categories of independent variables in the analysis based on a stepwise and forward model selection approach. The variable categories include (i) sociodemographic background; (ii) personalised psychosocial attributes (levels of HIV misconceptions, negative condom attitude, age at sexual debut, and resilience); and (iii) socially ascribed psychosocial experiences (everyday discrimination and pro-community attitudes). After preliminary univariate and bivariate analyses, we used a hierarchical linear regression model (HLM) to predict levels of HIV misconceptions while controlling for the effect of the city of residence. Results. More than 50% of participants in all study sites were aged 20–49 years, married, and have undergone a college or university undergraduate education. Yet, a significant proportion (27.2%) held varying levels of misconceptions about HIV. In those with misconceptions, the two most common misconceptions were: (i) people are likely to get HIV by deep kissing, putting their tongue in their partner’s mouth, if their partner has HIV (40.1%); and (ii) taking a test for HIV one week after having sex will tell a person if she or he has HIV (31.6%). Discrimination (β = 0.23, *p* < 0.05, 95% CI = 0.01, 0.46), negative condom attitudes (β = 0.07, *p* < 0.05, 95% CI = 0.01, 0.12), and sexual debut at an older age (β = 0.06, *p* < 0.05, 95% CI = 0.01, 1) were associated with more HIV misconceptions. Being born in Canada (β = −0.96, *p* < 0.05, 95% CI = −1.8, −0.12), higher education (β = −0.37, *p* < 0.05, 95% CI = −0.52, −0.21), and being more resilient (β = −0.04, *p* < 0.05, 95% CI = −0.08, −0.01) were associated with fewer HIV misconceptions. Conclusion and recommendations. HIV misconceptions are still common, especially among HBM. These misconceptions are associated with structural and behavioural factors. We recommend structural and policy-driven interventions that promote more accessible and equity-driven healthcare, education, and social integration of HBM in Ontario. We also recommend building capacity for collective resilience and critical health and racial literacy as well as creating culturally safe spaces for intergenerational dialogues among HBM in their communities.

## 1. Introduction

According to the Public Health Agency of Canada, compared to other provinces, Ontario had the highest number of people living with HIV in 2020 [1]. In addition, 25% of new HIV cases in Ontario can be attributed to people of African, Caribbean, and Black descent [2]. Although the Black population has a high prevalence of HIV [3], about 85% of those diagnosed are on antiretroviral therapy (ART), and about 96% those on ART have a suppressed viral load [1,4]. Prevention, early diagnosis, treatment, and linkage to care are core to public health HIV responses. However, HIV misconceptions pose a great challenge to effective HIV prevention and responses. Black people in Canada are disproportionately affected by HIV. In 2020, 19.7% of all the new HIV diagnoses in Canada were be attributed to Black men [5], yet Black people make up only 4.26% of the population. Given the relatively higher prevalence rate, any persistent misconceptions need to be addressed among Black men [4]. Underlying misconceptions that Black men might have about HIV mediated by social determinants likely contribute to the disproportionate impact of HIV among Black men and their communities. HIV misconceptions are defined as “…ideas and beliefs about the transmission of HIV that are proven to be factually inaccurate” [6]. Misconceptions often function as barriers to new learning and pose challenges to HIV prevention education. People with misconceptions tend to hold onto their prior knowledge or beliefs established through flawed reasoning or misinterpretation of information [7,8]. Habitual thinking makes it difficult for people to accept new knowledge, and they often revert to their established misconceptions even after being provided with the correct knowledge [9]. Hence, identifying contributing factors to misconceptions about HIV is essential to address and improve HIV prevention among Black men and communities.

Some common misconceptions of HIV among HBM in Ontario include beliefs that heterosexual men have a lower risk of getting HIV [10]. Some also believe that only people with substance use get HIV/AIDS [10]. Others have the belief that getting HIV tests could emasculate heterosexual men [11,12,13]. Conspiracy beliefs about HIV have also been found in a study of African American men [14]. Some participants believed that HIV is a virus produced or spread by the public health authorities; some believe that HIV is deliberately used to control Black people, or that HIV is a form of genocide against Black people [15]. Some believed that poor Black people were deliberately prevented from obtaining the cure for HIV [15]. In contrast, others thought that Black people were being used as guinea pigs to test the new drugs, or that the medications were deliberately given to them to infect them with AIDS [15].

Misconceptions have serious negative consequences on the health of HBM and communities because they can inadvertently impact their health-seeking behaviours [16]. For example, Bogart et al. showed in their study that certain misconceived beliefs among heterosexual South African men and women affected their intention to use a condom, an effective tool to prevent HIV [16]. Equally, misconceived ideas about HIV can reinforce social stigma against people living with HIV and deter health-seeking behaviours related to HIV [10,17,18] Stigma creates barriers for people to get tested, which is a significant challenge for the prevention of HIV among HBM in Ontario [10]. Furthermore, these misconceptions may reinforce the belief that condom use is not necessary, resulting in unprotected sex and a higher risk of HIV [19].

Although misconceptions are difficult to address by merely providing accurate HIV knowledge, a critical step is to identify the underlying beliefs and inaccurate knowledge before making effort to address them [9]. In this paper we describe the types of misconceptions about modes of HIV transmission held by some HBM in Ontario. Furthermore, we also identified the social determinants of HIV misconceptions, that is, the protective versus risk factors for HIV misconceptions among HBM in Ontario. Our goal is to inform evidence-based strategies for strengthening HIV prevention and stigma reduction among HBM and communities in Ontario.

## 2. Methods

This paper reports findings of the weSpeak study which was carried out in four cities (Ottawa, Toronto, London, and Windsor) in Ontario [20]. A total of 866 participants completed the questionnaire: Ottawa (n = 210), Toronto (n = 343), London (n = 157), and Windsor (n = 156). We estimated that a sample of 866 would be adequate for the study as it is more than the minimum sample size (384) needed to have 95% confidence level for a margin of error of 5% for the total population of Black people in Ontario. Venue-based sampling supported with the innovative use of peer research associates (PRAs) facilitated participant recruitment from difficult-to-reach areas of the Black community. PRAs are members of the Black communities with experiences in working or collaborating with diverse stakeholders (e.g., community health organisations, Black churches, Black business, etc.) and were trained by the research investigation team and mentored by the research coordinator. PRAs’ training addressed areas such as ethical considerations, survey administration, critical health and racial literacy, and social determinants of health in Black communities.

For this paper, three categories of data were drawn from the survey results, including: (i) sociodemographic background (city of residence, country of birth, age, marital status, educational attainment, employment status, and religious status); (ii) personalised psychosocial attributes (levels of HIV misconceptions, negative condom attitude, age at sexual debut, and resilience); and (iii) socially ascribed psychosocial experiences (everyday discrimination and pro-community attitudes). Measures of these variables have been fully expatiated in the weSpeak study protocol [20]. The outcome variable, HIV misconception, was measured as the number of statements on the HIV Knowledge Questionnaire with incorrect answers. Misconception scores ranged from 1 to 18. We used univariate analyses to generate frequency distribution tables of the predictor and outcome variables. The bivariate analyses were used to study the correlation of each of the predictor variables with the outcome variable (HIV misconception). The results of the bivariate analyses guided the selection of variables for the multivariate analysis or the hierarchical linear regression model (HLM). After the univariate and bivariate analyses, we used the HLM to predict levels of HIV misconceptions while controlling for the effect of the city of residence. We obtained the best fit model with a stepwise and forward selection approach by which most significant variables were included and least significant variables excluded in sequence, starting with a model with the intercept alone. In the model selection process, we realised that inclusion of city of residence as a predictor reduced the model fit and it had no significant independent association with HIV misconceptions, therefore we chose the model without city of residence as the lead model. After variable selection, the final model was achieved in a two-step process. We first included the sociodemographic factors to estimate their differential effect on HIV misconception, and then we included the protective and risk factors of HIV misconceptions in the second step. Significance of explanatory variables was decided at *p* < 0.05. The explanatory variables that were used in the analysis include: (i) sociodemographic factors: country of birth dummy (Canada = 1, other countries = 0), employment status (employed full time = 1, others = 0), religion (non-religious = 1, otherwise = 0), and education (more than high school = 1, high school or lower = 0); (ii) personalised psychosocial factors: age at sexual debut (years), negative condom attitudes (scale score, Cronbach α = 0.89), and resilience (scale score, Cronbach α = 0.76–0.84) [21,22]; and (iii) socially ascribed psychosocial factors: discrimination (scale score) and positive pro-Black community attitudes (scale score, Cronbach α = 0.81) [23]. Measures of these psychosocial variables have been fully described in previous studies [19,20,24].

## 3. Results

Sociodemographic statistics of the research participants are shown in Table 1. Precisely 39.6% of the participants reside in Toronto and the greater Toronto area, 24.3% live in Ottawa. Most of the participants were Black immigrants (i.e., born abroad), with percentages ranging from 58.4% in Windsor to 76% in London. Additionally, most participants were in the middle age groups of 20–49 years. More than half (51.28% to 60.5%) of participants in all study sites were single. Similarly, over half of participants (50.3% to 60.7%) have undergone a college or university undergraduate education. A large majority of participants (68.4% to 83.3%) self-identified as Christians. Most participants were employed in all sites; 39.9% to 60.9% were in full-time employment. However, significant percentages of participants were unemployed in all cities (21.2% to 42.6%).

The mean pro-Black community attitudes scores were very close in all cities, with Ottawa having the highest value of 16.3 out of a maximum of 25. Similarly, mean resilience scores were very close in all cities, and London recorded the highest mean score (58.9) per maximum score (80). Mean discrimination scores were approximately 20 per maximum (30) in all cities except Windsor (17.9). Mean negative condom attitudes scores ranged from 24.6 to 27.2 per maximum (45) with the highest mean score recorded in Windsor (27.2). Average age at sexual debut was greater in London (19 years) and Windsor (23.5 years) than in Toronto (17.1 years) and Ottawa (17.8 years).

Table 1 also shows correlation coefficients (β) of each of the variables with the outcome without accounting for the effects of other predictors on the outcome (HIV misconceptions). The β estimates are therefore unadjusted for the effects of other variables on HIV misconceptions. The adjusted model provides results that are relevant for selecting variables used in the adjusted model (HLM). Results of the unadjusted model show that belonging to the age group of 15–19 years and having negative condom attitudes each correlated with increased HIV misconceptions. On the other hand, being married, being a Christian, being in full-time employment, and being resilient each correlated with fewer HIV misconceptions. However, when all these significant variables from the unadjusted model were entered into the adjusted model, some of them were dropped and others were entered until model fitness was attained.

Table 2 describes percentages of the HBM with various levels of HIV misconceptions. Most (63.2%) of the Black men were highly knowledgeable about HIV, but a significant proportion (27.2%) of the HBM in all sites held varying levels of misconceptions about HIV. Precisely 23.1% of the HBM in all sites had misconceptions on 1–5 items on the 18-item HIV knowledge scale, 3.2% had misconceptions on 6–10 items, and 0.9% had misconceptions on more than 10 items.

**Figure 1:**
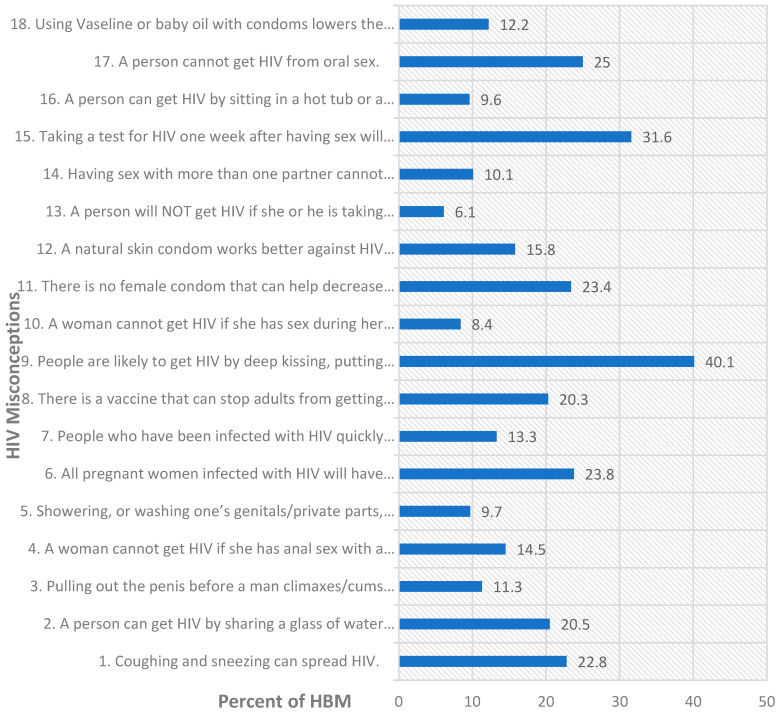
Percentages of HBM in all study sites with misconceptions on each item on the HIV-KQ-18.

Figure 1 presents the 18 HIV misconceptions item by item versus the percentages of HBM who held each misconception. The most common misconceptions based on the percentages of HBM who held them were: (i) people are likely to get HIV by deep kissing, putting their tongue in their partner’s mouth, if their partner has HIV (40.1%); (ii) taking a test for HIV one week after having sex will tell a person if she or he has HIV (31.6%); (iii) a person cannot get HIV from oral sex (25%); (iv) all pregnant women infected with HIV will have babies born with AIDS (23.8%); (v) there is no female condom that can help decrease a woman’s chance of getting HIV (23.4%); coughing and sneezing can spread HIV (22.8%); (vi) a person can get HIV by sharing a glass of water with someone who has HIV (20.5%); and (vii) there is a vaccine that can stop adults from getting HIV (20.3%). Table A1 provides the HIV misconceptions and accurate HIV knowledge side by side alongside explanatory notes. Analyses of HIV misconceptions by city of residence indicate that there were relatively few HBM with some level of misconception in Toronto (25.4%), the most in Ottawa (28.6%) and in London (28.6%), but slightly fewer in Windsor (28.2%).

Table 3 shows results of hierarchical linear modelling to determine factors of HIV misconceptions among the HBM. All the social determinants of health included in the model jointly contributed 20% (adjusted *R*^2^ = 0.2, *p* < 0.001) to variation in the levels of misconception about HIV. Results by independent predictors show that discrimination (β = 0.23, *p* < 0.05, 95% CI = 0.01, 0.46), negative condom attitudes (β = 0.07, *p* < 0.05, 95% CI = 0.01, 0.12), and sexual debut at an older age (β = 0.06, *p* < 0.05, 95% CI = 0.01, 1) were associated with more HIV misconceptions. Being born in Canada (β = −0.96, *p* < 0.05, 95% CI = −1.8, −0.12), higher education (β = −0.37, *p* < 0.05, 95% CI = −0.52, −0.21), and being more resilient (β = −0.04, *p* < 0.05, 95% CI = −0.08, −0.01) were associated with fewer HIV misconceptions.

## 4. Discussion

We discuss the findings regarding risk factors and protective factors of HIV misconceptions among HBM alongside previous literature on these topics.

### 4.1. Risk Factors of HIV Misconceptions

This study found that experiences of all forms of everyday discrimination are associated with higher levels of HIV misconceptions among HBM. This is consistent with previous studies that reported that discrimination in all forms contributes to the formation of HIV misconceptions among HBM either directly or indirectly. For instance, a study attributed HIV misconceptions among minority groups to trust issues developed from their experiences of discrimination [25]. Another study established a positive correlation between experiences of prejudice and HIV misconceptions, confirming that discrimination experiences contribute to HIV misconceptions [26]. An example of a misconception is the idea that HIV was produced in a laboratory to target minority groups [26]. Conversely, some studies have shown that HIV misconceptions can contribute to discrimination, e.g., homophobic beliefs that only gay men are at risk of HIV, thus creating a cycle in which discrimination perpetuates HIV misconceptions, misconceptions worsen HIV-related stigma, the stigma demotivates HIV testing, low testing rates then reduce treatment and prevention and ultimately perpetuate discriminative behaviours and attitudes towards those with the disease [14,17,18], and the cycle continues.

In this study, we established a positive correlation between HIV misconceptions and negative condom attitudes., Other research has linked HIV conspiracy beliefs with negative attitudes among Black men towards condoms, and hence they are associated with lower odds of consistent condom use [27,28]. For example, studies conducted among African men showed that misconceptions (e.g., witchcraft contributes to HIV transmission, condoms spread HIV/AIDS, vitamins cure AIDS, etc.) were correlated with negative condom attitudes [16,29,30,31]. Hence, negative condom attitudes and misconceptions are strong dual determinants of HIV vulnerability that need to be addressed together.

Against our a priori expectations, we found a positive correlation between age at sexual debut and HIV misconceptions, indicating that HBM who had their sexual debut at an older age (e.g., at above 18 years) had more HIV misconceptions. This finding is contradictory to earlier studies with non-migrant Black men in Africa, which found that early sexual debut was associated with more HIV misconceptions [26,32,33,34,35]. While this result needs to be further explored as it is counterintuitive, there are numerous probable explanations. In Ontario, most young people under the age of 18 attend secondary schools, where sexual health education is part of the curricula. Young HBM who had their sexual debuts while attending secondary schools were more likely to pay attention to sexual health education and therefore had fewer HIV misconceptions. Young HBM who had their sexual debuts after secondary school had less access to HIV prevention information. In addition, the greater misconceptions might be related to mistrust of information from the healthcare system due to systemic barriers that older sexual debutants may have faced over the years.

This study shows that Canadian-born HBM have more HIV misconceptions than their immigrant counterparts, indicating that country of birth may influence levels of HIV misconceptions. This finding may be explained by the evidence from community discussions suggesting that HIV is not a priority issue for public health education in Canada compared to endemic countries in Africa. In addition, existing dominant messages still focus on gay White men and racialised women, but not HBM. Additionally, studies have shown that first-generation immigrants tend to have better health practices than other generations based on their stronger connection and a sense of responsibility towards their family in their pre-migration countries [12,26,34,35]. There is also strong evidence on the healthy immigrant effect, i.e., new immigrants tend to be healthier than those who were born in Canada or have settled in Canada longer [12,36]. Prior to immigration, new immigrants often undergo a health test including a pre-immigration HIV test in Canada, to ensure they are in good health [12,37]. However, long-standing experiences of discrimination and other structural barriers by older immigrants and their later generations predispose them to mistrust the healthcare system and develop misconceptions and poor health-seeking behaviour.

### 4.2. Protective Factors of HIV Misconceptions

Despite existing misconceptions among HBM, consistent with previous work, we found education to be a strong mediator of HIV misconceptions whereby educated people were less likely to express any misconceptions [10,12,14]. Increased access to formal education is therefore an indirect but critically essential intervention to reduce the spread of HIV among HBM and communities. Statistics from the Canadian 2016 census indeed show the need for increased access to education among Black men. For example, fewer young Black men had attained post-secondary education than men of other races, and 17% of Black men had obtained a university degree compared to 26% of men from other races [38,39,40,41]. Worse still, young Black men were twice as likely not to be employed regardless of having a university degree [37]. This situation further demotivates upcoming young Black men from seeking university education.

Beyond formal education, inadequate and incorrect HIV knowledge plays a significant role in producing HIV misconceptions. HIV education is therefore an important tool to curb misconceptions about HIV [10]. It enables people to engage in meaningful discussions and make better-informed decisions necessary for HIV prevention practices [38,39]. Culturally relevant and inclusive HIV education in Black communities fostered by trust, cohesion, peer support, and participation can promote health-enhancing knowledge and not misconceptions. When HIV education is delivered in safe spaces, HBM can share experiences and collaborate in learning effective strategies to promote and maintain their individual and collective health and reduce HIV vulnerabilities in Black communities. Safe and inclusive spaces are important because they offer a stigma-free and racism-free environment needed to promote community dialogue and effective HIV prevention and care messages [40]. In such spaces: (i) lived experiences of HIV/AIDS are honoured and not stigmatised, (ii) community-driven HIV prevention programmes can be implemented without barriers, and (iii) intergenerational dialogues on HIV prevention, diagnoses, treatment, and linkage to care are valued [41].

We also found that HBM who reported higher levels of individual and collective resilience had fewer HIV misconceptions. This is expected because resilience represents the intertwined personal and collective capacity to solve problems and thrive in the face of adversity [41,42]. Building strong and appropriate social networks, close connections with family and friends, and other resources are vital protective factors for resilience [43,44]. Connecting with social supports in family, community, and healthcare settings provides people with a sense of belonging and trust that supports them to take up correct information and let go of misconceptions. Studies have shown that stigma against people living with HIV, associated with misconceptions, has been mitigated by these resilience-related protective factors [45,46]. Collective resilience has been found to be associated with lower overall HIV risk and increased use of health services [47,48,49,50] which are determinants of lower levels of HIV misconceptions.

## 5. Conclusions and Recommendations

Misconceptions about HIV are still found among a small but significant population of HBM. Structural determinants such as experiences of discrimination, especially within the healthcare system, may be associated with more misconceptions, while the extent of integration (e.g., born in Canada versus immigrant) and levels of formal education may be associated with reduced misconceptions. In addition, behavioural factors such as negative condom attitudes are important risk factors for HIV misconceptions, just as personalised resilience is a strong protective factor against HIV misconceptions. We propose a wholistic approach to addressing HIV misconceptions including structural and policy-driven interventions that promote more accessible and equity-driven healthcare, education, and social integration of HBM in Ontario. We also recommend building capacity for collective resilience and critical health and racial literacy as well as creating culturally safe spaces for intergenerational dialogues among HBM in their communities.

## Figures and Tables

**Table 1:** Descriptive analysis of independent variables: socioeconomic and psychosocial factors.

Variables	Toronto n (%)	Ottawa n (%)	London n (%)	Windsor n (%)	β (Unadjusted Model)
City of residence	343 (39.6)	210 (24.3)	157 (18.1)	156 (18.0)	−0.05
Country of birth					
Born in Canada	98 (29.62)	60 (28.85)	37 (24.0)	64 (41.6)	0.11
Born abroad	238 (70.8)	148 (71.15	117 (76.0)	90 (58.4)	ref
Total valid responses	336 (100)	108 (100)	154 (100)	154 (100)	
Age categories (in years)					
15–19	27 (7.9)	42 (20.00)	22 (14.0)	11 (7.05)	3.78 *
20–29	94 (27.4)	66 (31.43)	47 (29.9)	57 (36.54)	1.14
30–39	100 (29.1)	50 (23.81)	42 (26.8)	32 (20.51)	0.65
40–49	60 (17.5)	38 (18.1)	25 (15.9)	15 (9.61)	1.18
50–59	27 (7.9)	4 (1.9)	17 (10.8)	29 (18.59)	0.65
60–64	19 (5.5)	6 (2.86)	3 (1.91)	6 (3.85)	1.63
65 and older	16 (4.7)	4 (1.9)	1 (0.6)	6 (3.85)	ref
Total valid responses	343 (100)	210 (100)	157 (100)	156 (100)	
Marital status					
Single	151 (53.0)	115 (54.8)	81 (60.5)	80 (51.28)	−0.13
Married	115 (40.3)	60 (28.6)	46 (34.3)	44 (28.21)	−0.93 *
Others	19 (6.7)	35 (16.6)	7 (5.2)	32 (20.51)	ref
Total valid responses	285 (100)	210 (100)	134 (100)	156 (100)	
Education					
High school or lower education	118 (34.8)	64 (31.7)	44 (28.8)	36 (24.0)	3.22 **
College or university undergraduate	178 (52.5)	110 (54.4)	77 (50.3)	91 (60.7)	0.65
University graduate or professional degree	43 (12.7)	28 (13.9)	32 (20.9)	23 (15.3)	ref
Total valid responses	339 (100)	202 (100)	153 (100)	150 (100)	
Religious affiliation					
Muslim	39 (12)	32 (16.6)	14 (9.5)	9 (6)	−0.13
Christian	235 (71.9)	132 (68.4)	116 (78.9)	124 (83.3)	−1.28 *
African traditional	5 (1.5)	5 (2.6)	1 (0.7)	0 (0)	−1.13
Others	5 (1.5)	4 (2)	2 (1.4)	3 (2)	−0.46
None	43 (13.1)	20 (10.4)	14 (9.5)	13 (8.7)	Ref
Total valid responses	327 (100)	193 (100)	147 (100)	149 (100)	
Employment status					
Employed (full time)	177 (54.3)	120 (60.6)	59 (39.9)	92 (60.9)	−1.1 **
Employed (part time)	45 (13.8)	28 (14.1)	26 (17.5)	27 (17.9)	−0.71
Unemployed	104 (31.9)	50 (25.3)	63 (42.6)	32 (21.2)	Ref
Total valid responses	324 (100)	198 (100)	148 (100)	151 (100)	
Pro-Black community attitudes score (m ± SD)	15.7 ± 3.8	16.3 ± 4.0	15.9 ± 3.2	16.1 ± 3.2	−0.07
Resilience (m ± SD)	58.6 ± 9.5	57.4 ± 8.4	58.9 ± 8.0	56.8 ± 8.8	−0.07 ***
Everyday discrimination score (m ± SD)	20.4 ± 6.4	20.4 ± 6.1	20.3 ± 6.3	17.9 ± 7.9	−0.02
Negative condom attitudes (score)	25.4 ± 6.2	26.2 ± 5.8	24.6 ± 6.2	27.2 ± 6.0	0.08 **
Age at sexual debut (m ± SD)	17.1 ± 3.9	17.8 ± 6.3	19 ± 7	23.5 ± 15.4	0.03

* *p* < 0.05, ** *p* < 0.01, *** *p* < 0.001; β = correlation coefficient, m = mean, SD = standard deviation.

**Table 2:** Percentages of Participants with HIV Misconceptions versus those with HIV Knowledge across Cities.

Score Categories	Toronto (n = 343)	Ottawa (n = 210)	London (n = 157)	Windsor (n = 156)	All Sites (N = 866)
None (0)	3.5 (11.7)	3.3 (11.9)	3.8 (10.8)	2.6 (13.5)	3.3 (11.9)
(1–5)	22.2 (6.1)	22.9 (7.1)	22.3 (4.5)	26.3 (10.3)	23.1(6.8)
(6–10)	2.6 (17.2)	3.3 (22.9)	5.7 (16.6)	1.9 (15.4)	3.2 (18.1)
(>10)	0.6 (65.0)	2.4 (58.1)	0.6 (68.2)	0.0 (60.9)	0.9 (63.2)
Misconception (%)	25.4	28.6	28.6	28.2	27.2

Outside parentheses: % participants with HIV misconceptions; in parentheses: % participants with HIV knowledge. HIV misconceptions score = number of wrong responses on the HIV Knowledge Questionnaire. HIV knowledge score = number of correct responses on the HIV Knowledge Questionnaire.

**Table 3:** Social Determinants of HIV Misconceptions: Results of Hierarchical Linear Model.

Outcome: HIV Misconceptions (Score)	Model 1		Model 2	
Predictors:	β	95% CI	β	95% CI
Country of birth dummy (Canada = 1, other countries = 0)	−1.06 *	−1.9, −0.22	−0.96 *	−1.8, −0.12
Employment status (employed full time = 1, others = 0)	0.46	−0.27, 1.19	0.46	−0.24, 1.16
Religion (non-religious = 1, otherwise = 0)	0.39	−0.83, 1.61	0.78	−0.42, 1.98
Education (more than high school = 1, high school or lower = 0)	−0.35 ***	−0.51, −0.19	−0.37 *	−0.52, −0.21
Resilience (score)			−0.04 *	−0.08, −0.01
Discrimination (score)			0.23 *	0.01, 0.46
Negative condom attitudes (score)			0.07 *	0.01, 0.12
Positive pro-Black community attitudes (score)			0.05	−0.05, 0.14
Age at sexual debut (years)			0.06 *	0.01, 0.1
Model summary				
*R*^2^ change		0.14 ***		0.1 **
Adjusted *R*^2^		0.12 ***		0.2 ***

β = correlation coefficient, * *p* < 0.05, ** *p* < 0.01, *** *p* < 0.001.

## Data Availability

Data will be uploaded to a data repository in due course.

## Appendix A

**Table A1:** Misconceptions Versus Correct Knowledge about modes of HIV transmission.

S/N	Misconceptions	Correct Knowledge	Explanatory Notes
1	Coughing and sneezing can spread HIV.	Coughing and sneezing DO NOT spread HIV.	Because HIV is not airborne, it is not carried by tiny particles from sneezes and coughs [51].
2	A person can get HIV by sharing a glass of water with someone who has HIV.	A person CANNOT get HIV by sharing a glass of water with someone who has HIV.	HIV cannot be spread by sharing drinking glasses because science shows that the risk of getting it from saliva is extremely low.
3	Pulling out the penis before a man climaxes/cums keeps a woman from getting HIV during sex.	Pulling out the penis before a man climaxes/cums MAY NOT keep a woman from getting HIV during sex.	Although semen is one of the most common ways through which HIV is transmitted during sex, pre-ejaculatory fluid may also transmit HIV [52,53].
4	A woman cannot get HIV if she has anal sex with a man.	A woman can get HIV if she has anal sex with a man.	Anal sex is the riskiest type of sex for getting or transmitting HIV and being the receptive partner (bottom) makes it riskier than being the insertive partner (top) [54].
5	Showering, or washing one’s genitals/private parts, after sex keeps a person from getting HIV.	Showering, or washing one’s genitals/private parts, after sex CANNOT keep a person from getting HIV.	Surprisingly, a study showed that washing the penis immediately after increases the risk of acquiring HIV by uncircumcised men [55].
6	All pregnant women infected with HIV will have babies born with AIDS.	NOT ALL pregnant women infected with HIV will have babies born with AIDS.	If the woman is on antiretroviral therapy during pregnancy, she can prevent transmission of HIV to the unborn baby.
7	People who have been infected with HIV quickly show serious signs of being infected.	People who have been infected with HIV DO NOT quickly show serious signs of being infected.	Most (80%) people infected with HIV experience a short, flu-like illness that occurs 2–6 weeks after infection. After this, HIV may not cause any symptoms for several (10) years [56].
8	There is a vaccine that can stop adults from getting HIV.	There is NO VACCINE that can stop adults from getting HIV.	Scientists are still working to develop an HIV vaccine, and Durbin, a professor in international health, says there will not be one by 2030 [57].
9	People are likely to get HIV by deep kissing, putting their tongue in their partner’s mouth, if their partner has HIV.	People are NOT LIKELY to get HIV by deep kissing, putting their tongue in their partner’s mouth if their partner has HIV.	You cannot transmit HIV through closed-mouth or “social” kissing with someone who has HIV, except very rarely, when both partners have mouth sores and bleeding gums [54].
10	A woman cannot get HIV if she has sex during her period.	A woman CAN get HIV if she has sex during her period.	Studies show that heterosexual transmission of HIV and other STIs happens more easily during menstruation [58].
11	There is no female condom that can help decrease a woman’s chance of getting HIV.	There is a female condom that can help decrease a woman’s chance of getting HIV.	There are female condoms, and they are as effective at protecting against HIV as male condoms. Like the male condoms they are available in some drug stores, community health centers, and AIDS service organisations.
12	A natural skin condom works better against HIV than does a latex condom.	A natural skin condom DOES NOT work better against HIV than does a latex condom.	Latex condoms provide the best protection against HIV. Natural membrane (such as lambskin) condoms have small holes in them and do not block HIV and other STDs [59].
13	A person will NOT get HIV if she or he is taking antibiotics.	A person MAY GET HIV Even if she or he is taking antibiotics.	Taking antibiotics does not prevent HIV but they can prevent STIs [60]. More research is needed to estimate the efficacy of antibiotics in preventing HIV transmission [61]. However, PrEP or PEP taken as prescribed by a physician prevents HIV.
14	Having sex with more than one partner cannot increase a person’s chance of being infected with HIV.	Having sex with more than one partner can increase a person’s chance of being infected with HIV.	Having multiple sex partners who overlap in time increases one’s and one’s regular partner’s risk of getting HIV and STIs [62].
15	Taking a test for HIV one week after having sex will tell a person if she or he has HIV.	Taking a test for HIV one week after having sex WILL NOT tell a person if she or he has HIV.	A rapid antigen test carried out with blood from a finger detects HIV 18 to 90 days after exposure. An antigen lab test using blood from a vein detects HIV 18 to 45 days after exposure. A nucleic acid test detects HIV 10 to 33 days after exposure [63].
16	A person can get HIV by sitting in a hot tub or a swimming pool with a person who has HIV.	A person CANNOT get HIV by sitting in a hot tub or a swimming pool with a person who has HIV.	HIV does not survive in water or hot tubs, but it survives in some human fluids: blood, semen, pre-seminal fluid, rectum fluids, vaginal fluids, and breast milk [64].
17	A person cannot get HIV from oral sex.	A person can get HIV from oral sex.	Putting a mouth on the penis, vagina/vulva, or anus would normally not transmit HIV but ejaculating in a mouth with oral ulcers, bleeding gums, or genital sores and when having STIs increases the risk of getting HIV [62].
18	Using Vaseline or baby oil with condoms lowers the chance of getting HIV.	Using Vaseline or baby oil with condoms CANNOT lower the chance of getting HIV.	Using baby oil on a latex condom may cause the condom to break and increase the risk of getting HIV. However, one can use recommended water- or silicone-based lubricants [62].
